# Endothelial permeability following coronary artery bypass grafting: an observational study on the possible role of angiopoietin imbalance

**DOI:** 10.1186/s13054-016-1238-0

**Published:** 2016-03-06

**Authors:** Tobias Hilbert, Georg Daniel Duerr, Marwan Hamiko, Stilla Frede, Lynette Rogers, Georg Baumgarten, Andreas Hoeft, Markus Velten

**Affiliations:** Department of Anesthesiology and Intensive Care Medicine, University Medical Center Rheinische Friedrich-Wilhelms-University Bonn, Bonn, Germany; Department of Cardiovascular Surgery, University Medical Center Rheinische Friedrich-Wilhelms-University Bonn, Bonn, Germany; Center for Perinatal Research, The Research Institute at Nationwide Children’s Hospital, Columbus, OH USA

**Keywords:** Angiopoietin, CABG, Capillary leakage, Endothelial dysfunction, Postoperative mortality

## Abstract

**Background:**

Unresolved inflammation resulting in capillary leakage with endothelial barrier dysfunction is a major contributor to postoperative morbidity and mortality after coronary artery bypass graft (CABG). Angiopoietins (ANGs) are vascular growth factors, also mediating inflammation and disruption of the endothelium, thus inducing capillary leakage. We hypothesized that changes in the relative serum levels of ANG1 and ANG2 influence endothelial barrier function and perioperative morbidity after CABG.

**Methods:**

After approval and informed consent, serum samples (*n* = 28) were collected pre CABG surgery, 1, 6, and 24 h after aortic de-clamping. ANG1, ANG2, soluble ANG receptor TIE2 (sTIE2), and IL-6 serum concentrations were analyzed by ELISA. Human pulmonary microvascular endothelial cells (HPMECs) were incubated with patient serum and FITC-dextran permeability was assessed. Furthermore, ANG2 secretion of HPMECs was analyzed after incubation with IL-6-containing patient serum.

**Results:**

CABG induced an early and sustained increase of ANG2/ANG1 ratio (5-fold after 24 h compared to pre-surgery). These changes correlated with elevated serum lactate levels, fluid balance, as well as the duration of mechanical ventilation. Permeability of HPMECs significantly increased after incubation with post-surgery serum showing a marked shift of ANG2/ANG1 balance (18-fold) compared to serum with a less pronounced increase (6-fold). Furthermore, CABG resulted in increased IL-6 serum content. Pre-incubation with serum containing high levels of IL-6 amplified the ANG2 secretion by HPMECs; however, this was not influenced by blocking IL-6.

**Conclusions:**

CABG affects the balance between ANG1 and ANG2 towards a dominance of the barrier-disruptive ANG2. Our data suggest that this ANG2/ANG1 imbalance contributes to an increased postoperative endothelial permeability, likewise being reflected by the clinical course. The results strongly suggest a biological effect of altered angiopoietin balance during cardiac surgery on endothelial permeability.

## Background

Coronary artery disease (CAD) is a significant health threat with increasing prevalence and enormous socioeconomic consequences, and is the leading cause of morbidity and mortality in the western world [1]. In addition to percutaneous coronary intervention, coronary artery bypass graft (CABG) surgery remains the gold standard therapy, especially in patients suffering from diffuse coronary stenosis [2].

During CABG, global perfusion is routinely maintained by establishing a cardiopulmonary bypass (CPB), usually by means of a heart-lung machine (HLM), so-called on-pump surgery. In addition to the surgical trauma, perioperative myocardial injury, and global ischemia reperfusion injury, on-pump surgery commonly results in systemic inflammatory response syndrome (SIRS) [3]. Development of SIRS influences the postoperative morbidity and mortality and therefore contributes to prolonged ICU treatment after CABG [4, 5]. One fundamental characteristic of SIRS is the development of endothelial barrier dysfunction [6]. Activation of the endothelium induces myosin-mediated contractions of endothelial cells (ECs), leading to the formation of intercellular gaps and subsequent extravasation of fluids, resulting in hypovolemia, edema, and organ dysfunction such as acute renal failure, acute respiratory distress syndrome (ARDS), and cerebral edema [7–10].

**Fig. 1:**
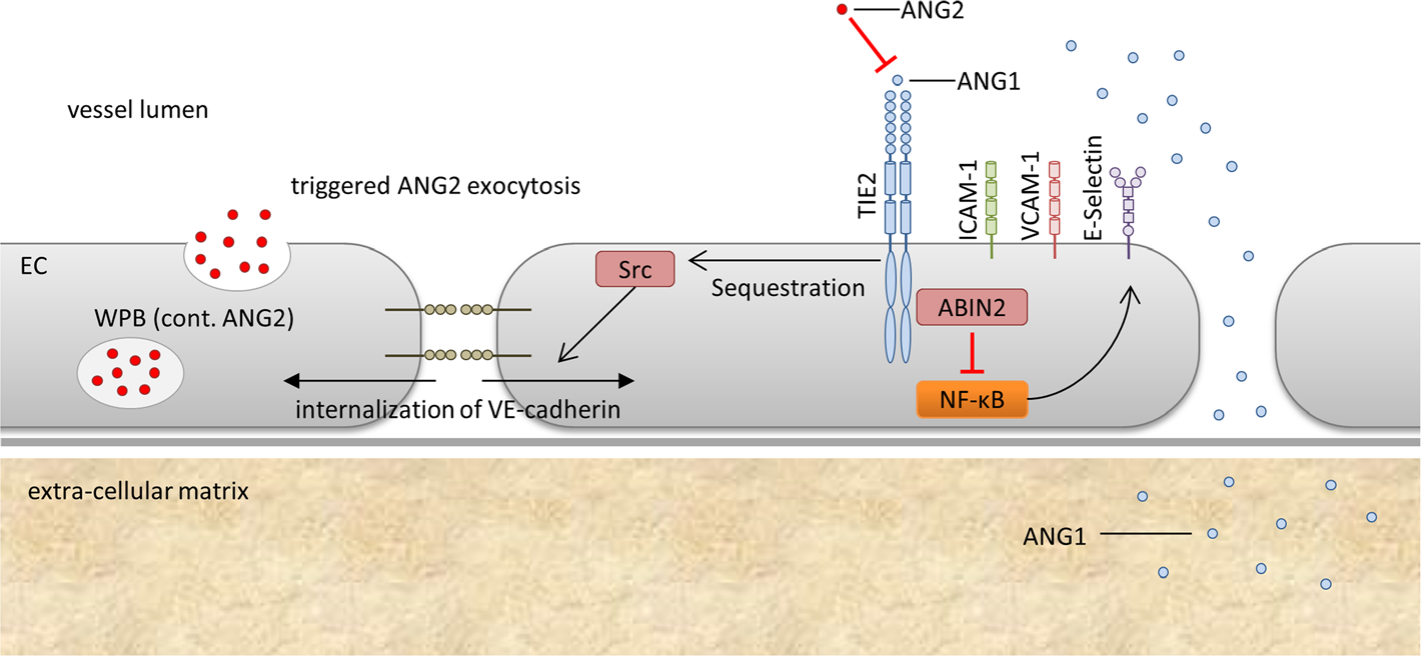
Angiopoietin signaling at the TIE2 receptor. Angiopoietin 1 (ANG1) is constitutively secreted by perivascular mural cells. Ligand-binding of (*ANG1*) to TIE2 induces sequestration of the tyrosine kinase Src and thus establishes stable expression of VE-cadherin on the surface of the endothelial cell. ANG2 is stored in Weibel-Palade (*WBP*) bodies and rapidly released upon triggering signals. Its binding to TIE2 abolishes ANG1-induced sequestration of Src, resulting in the internalization of VE-cadherin. Furthermore, sensitizing endothelial cells (*EC*) for ligands targeting nuclear factor-κB (*NF- κB*), such as vascular endothelial growth factor or tumor necrosis factor-α, results in the expression of adhesion molecules (selectines, intercellular adhesion molecule-1 (*ICAM*), vascular cell adhesion molecule-1 (*VCAM*)), which facilitates leukocyte adhesion and transmigration

Vascular endothelial growth factor (VEGF), initially termed vascular permeability factor, is linked to the development of endothelial barrier dysfunction after CABG [11]. Angiopoietins (ANGs) represent another prominent vascular growth factor system. Besides their importance for the development and maturation of the vasculature, ANGs mediate activation, inflammation, and disruption of the endothelium [12–16]. ANG1, continuously secreted by perivascular mural cells, exerts stabilizing and anti-apoptotic effects on the endothelium by specific binding to the TIE2 receptor (Fig. 1) [17, 18]. This ensures endothelial integrity and prevents hyper-permeability and the trans-endothelial passage of inflammatory cells [12, 19]. In contrast, ANG2 is stored preformed in intracellular vesicles of the EC, the Weibel-Palade bodies (WPB) [20]. Distinct stimuli (hypoxia, trauma, endogenous substances such as thrombin or histamine) trigger its rapid exocytosis [20–22]. Following secretion, ANG2 competes with ANG1 for binding to the TIE2 receptor, mediating opposite effects: disruption, hyper-permeability, and pro-inflammation. ANG2 was identified as a serum marker of endothelial barrier dysfunction, and a shift in the relative balance between ANG2 and ANG1 unequivocally contributes to the collapse of the endothelial barrier in vitro and in vivo [13, 23–25].

We hypothesized that CABG induces substantial alterations in ANG1 and ANG2 serum levels and that these changes influence endothelial barrier function and the post-surgery course.

## Methods

### Study design

This observational study was conducted at the cardiac-surgery suite and ICU at the University Medical Center Bonn, Germany, in accordance with the Declaration of Helsinki and after approval by the institutional revenue board (IRB) at the Rheinische Friedrich-Wilhelms-University Bonn (protocol number 011/13). Inclusion criteria were age >18 years of age, elective on-pump CABG surgery, and written informed consent. The following patients were excluded from the study: those with simultaneous valve replacement, emergency procedures (including acute myocardial infarction within 48 h prior to surgery), previous major surgery during the last 2 weeks, pre-existing infection, malignancy, pre-existing renal dysfunction, or pregnancy.

After invasive blood pressure assessment was established, anesthesia was induced by intravenous (i.v.) administration of etomidate, sufentanil, and rocuronium and was further maintained using isoflurane and continuous sufentanil infusion. Corticoids were not given to any of the enrolled patients during the observation period. After intubation and insertion of a central venous line, median sternotomy was performed in apnea. Before starting the CPB, a heparin bolus (500 IU/kg body weight) was administered to obtain an activated clotting time >400 s. After cannulation of the ascending aorta and the right atrium, CPB was established using a roller pump for non-pulsatile flow (Advanced Perfusion System 1, Terumo Corporation, Tokyo, Japan) and a membrane oxygenator (Quadrox-I Adult, Maquet Getinge Group, Rastatt, Germany). The HLM system was primed with 1200 ml of crystalloid infusion solution (Jonosteril, Fresenius Kabi, Bad Homburg vor der Höhe, Germany). After full HLM support was installed, cardiac arrest was induced by administration of Calafiore warm blood cardioplegia and maintained by repetitive re-infusion into the aortic root in 20-minute intervals. The aorta was then cross-clamped. Mild hypothermia was introduced (34 °C). The left or right internal mammary artery (LIMA, RIMA) and the saphenous vein of either leg was used as graft. Upon finishing the distal anastomosis of all bypass grafts, the patients were re-warmed to 36.5 °C and, after de-clamping of the aorta and re-installing normal antegrade perfusion, were weaned from HLM. Thereafter, heparin was antagonized using protamine in an equal dose to that of heparin. All patients were transferred to the ICU for postoperative care and therapy.

### Collection of blood samples and clinical data

Blood (10 ml) of was drawn before the beginning of surgery (preop) and 1, 6, and 24 h after aortic de-clamping. Samples were immediately centrifuged (3,000 rpm, 4 °C, 10 minutes), and serum aliquots were stored at −80 °C for subsequent analysis.

The following clinical data were recorded during the perioperative period:

- duration of surgery (incision - suture) and CPB
- aortic cross-clamp and reperfusion times
- fluid balance (calculated once per hour)
- duration of mechanical ventilation (begins with intubation after initial induction of general anesthesia and ends with first extubation on ICU)
- length of stay (LOS) in the ICU
- LOS in the hospital

All data were collected during an observation period of up to 60 h after admission to the ICU. Clinical laboratory parameters were assessed on each patient at ICU admission and every 6–12 h during ICU treatment.

### Detection of ANG1, ANG2, soluble TIE2, and pro-inflammatory cytokines

Detection of ANG1, ANG2, and soluble TIE2 (sTIE2) levels in serum samples was performed using enzyme-linked immunosorbent assay (ELISA) (R&D systems, Wiesbaden-Nordenstadt, Germany) according to the manufacturer’s protocol. The ratio between ANG2 and ANG1 (ANG2/ANG1 balance) was calculated using the formula:

ANG2/ANG1 ratio = ANG2 (ng/ml)/ANG1 (ng/ml).

TNF-α, interleukin (IL)-1β, and IL-6 levels were determined using ELISA (BD Biosciences, San Jose, CA, USA) and expressed as pg/ml serum. All experimental analyses were performed in triplicates. Out of these results, the mean value was calculated and used for further statistical analyses. Serum samples obtained early (1 h) after aortic de-clamping were compared to clinical parameters describing potentially harmful influences of surgery and CPB, to detect possible causal relationships with changing angiopoietin and cytokine levels. To evaluate a potential influence of changing angiopoietin levels on the further clinical course, we compared clinical outcome data with serum samples obtained during the further postoperative ICU stay (6 and 24 h after CABG).

### Cell culture

Cryopreserved pooled human pulmonary microvascular endothelial cells (HPMECs) and the recommended ECGM-MV (endothelial cell basal medium (EBM-MV)) with cell-specific supplement) were purchased from PromoCell (Heidelberg, Germany). Cells of less than 6 passages were cultured in medium until 80–90 % confluence. For sub-cultivation, cells were washed, harvested using Accutase™ solution (Sigma-Aldrich, Taufkirchen, Germany), and seeded in ECGM-MV into the corresponding cell culture plastics depending on the individual corresponding assay. Cells were cultured at 37 °C in a 5 % CO_2_ humidified atmosphere. All culture plastics were coated with 0.2 % gelatin in phosphate-buffered saline (PBS) before use.

### Assessment of endothelial permeability

Transwell™ permeable supports (0.33-cm^2^, 0.4-μm pore size, polycarbonate membrane; Corning, Tewksbury, MA, USA) were coated with gelatin and equilibrated with medium. Subsequently, HPMECs in ECGM-MV were seeded into the upper compartment in high density and cultured to form a confluent monolayer. Culture medium was changed every other day and particular attention was paid not to abolish the cell layer integrity.

After 3 days, supernatant was replaced by fresh culture medium supplemented with sterile (0.2 μm)-filtered preop or 24-h serum, respectively. Based on previous dose titrations a 7.5 % serum supplement was the optimal concentration for these experiments. Control cells were supplemented with 7.5 % fetal calf serum (FCS). After 30-minute incubation, fluorescein isothiocyanate (FITC)-conjugated dextran (70 kiloDalton (kDa), final concentration 1 mg/ml; Sigma-Aldrich, St. Louis, MO, USA) was added to the upper compartment and the inserts were transferred into pre-warmed assay buffer (4-(2-hydroxyethyl)-1-piperazineethanesulfonic acid (HEPES)-buffered Hank’s balanced salt solution (HBSS), pH 7.4) on a magnetic stirrer (400 rpm). After an initial equilibration period of 15 minutes, thrombin (final concentration 1 IU/ml; Sigma-Aldrich, St. Louis, MO, USA) was added to the upper compartment to induce endothelial permeability. Control cells were treated with assay buffer. A 50-μl sample was taken from the lower compartment after 5, 10, 15, 30, and 60 minutes and was replaced by an equivalent volume of fresh assay buffer. Fluorescence was measured using a fluorescent plate reader (excitation 492 nm, emission 518 nm; Modulus II Microplate Multimode Reader, Promega, Fitchburg, WI, USA). Concentration of dextran was calculated using an experimentally derived standard curve and the flux over the membrane was calculated. Thrombin-induced hyper-permeability was expressed as times fold of the average basal flux during the initial 15-minute equilibration period before thrombin addition.

### Determination of ANG2 secretion by HPMECs

HPMECs were seeded into 96-well flat-bottom plates and cultured in ECGM-MV. At 80–90 % confluence, the supernatant was replaced by fresh culture medium supplemented with sterile (0.2 μm-)filtered preop or 1-h serum, respectively. Based on previous dose-response titrations a 5 % serum supplement was the optimal concentration for these experiments. Control cells were supplemented with 5 % FCS. In additional experiments, cells were incubated with an inhibiting anti-human-IL-6 antibody (1 μg/ml; BioLegend, San Diego, CA, USA). After 24 h incubation, the supernatant was discarded and 55 μl of fresh EBM-MV was added. WPB exocytosis and thus, ANG2 secretion was triggered by adding thrombin (1 IU/ml) to the wells. After 1 h the supernatant was collected and ANG2 concentration was determined by ELISA (R&D systems, Wiesbaden-Nordenstadt, Germany).

### Statistics

Statistical analysis and visualization of data was performed using GraphPad PRISM 5 (La Jolla, CA, USA). Unless otherwise stated, graphs represent median with percentile 25–75 (Q_1_–Q_3_). Values lying outside ± 2.5 times the median absolute deviation around the median were identified as outliers and thus excluded (according to the method presented by Leys et al.) [26]. The significance of time-dependent changes in serum data was tested using Kruskal–Wallis one-way analysis of variance followed by Dunn’s post-hoc test. Results of the in vitro experiments are presented as mean ± standard error of the mean (SEM), and the two-tailed (or one-tailed, if appropriate), unpaired Student’s *t* test was used to compare the different groups. Pearson’s correlation test was used to analyze association between serum data and clinicopathologic variables obtained at the same time. The alpha level was set at 5 %.

## Results

### Basic patient characteristics

A total of 28 consecutive patients were included in the study. Two patients were discharged from ICU before the end of the 24-h period after aortic de-clamping, and sample acquisition was not continued on the peripheral ward, resulting in missing samples for the 24-h time point (T_24_) for these two patients. Eighty-six percent were male and the median age was 72 years with a range from 55 to 83 years. The median body mass index (BMI) was 26.8 kg/m^2^. The median CPB time was 110 minutes, the median aortic cross-clamp time was 68 minutes, and the median time for reperfusion was 33 minutes. The median total procedure time (cutting to suture) was 279 minutes. The median ICU LOS was 23.9 h and the median hospital LOS was 14 days. No patient showed clinical signs of wound infection, required repeat surgery or died within 30 days post surgery (Table 1).

**Table 1:** Basic patient characteristics, intraoperative and postoperative details

Characteristics		
Male sex	Number (percentage)	24 (86)
Age, years	Median (range)	72 (55–83)
Body mass index, kg BW/m^2^	Median (range)	26.8 (19.6–37.2)
Cardiovascular status, kidney		
Ejection fraction, %	Median (range)	55 (22–85)
NYHA status	Median (range)	2 (1–3)
Arterial hypertension	Number (percentage)	28 (100)
Carotid stenosis	Number (percentage)	8 (29)
PAOD	Number (percentage)	1 (4)
Serum creatinine, mg/dl	Median (Q_1_–Q_3_)	1 (0.85–1.1)
Details of procedure		
Cutting-suture time, minutes	Median (Q_1_–Q_3_)	279 (233–291)
CPB time, minutes	Median (Q_1_–Q_3_)	110 (98–124)
Aortic cross-clamp/reperfusion time, minutes	Median (Q_1_–Q_3_)	105 (92–116)
Cardioplegic solution, ml	Median (Q_1_–Q_3_)	710 (700–732)
Number of grafts	Median (range)	4 (1–4)
Number of intraoperative. transfused RBC concentrates	Median (range)	0 (0–4)
Postoperative details		
PaO_2_/FiO_2_ ratio at admission to ICU	Median (Q_1_–Q_3_)	244 (195–326)
PaO_2_/FiO_2_ ratio after 6 h	Median (Q_1_–Q_3_)	333 (251–383)
PaO_2_/FiO_2_ ratio after 24 h	Median (Q_1_–Q_3_)	419 (295–497)
Lactate at admission to ICU, mmol/l	Median (Q_1_ − Q_3_)	1.1 (0.9–1.74)
Lactate after 6 h, mmol/l	Median (Q_1_–Q_3_)	1.28 (1.07–1.71)
Lactate after 24 h, mmol/l	Median (Q_1_–Q_3_)	1.3 (1.07–1.43)
Norepinephrine at admission to ICU, μg/kg BW*minute	Median (Q_1_–Q_3_)	0.03 (0–0.09)
Norepinephrine after 6 h, μg/kg BW*minute	Median (Q_1_ - Q_3_)	0.03 (0 − 0.06)
Norepinephrine after 24 h, μg/kg BW*minute	Median (Q_1_–Q_3_)	0 (0–0)
Hgb at admission to ICU, mmol/l	Median (Q_1_–Q_3_)	11.2 (10.8–12.7)
Hgb after 6 h, mmol/l	Median (Q_1_–Q_3_)	10.7 (10–11.5)
Hgb after 24 h, mmol/l	Median (Q_1_–Q_3_)	9.8 (9.3–10.5)
24 h crystalloid fluid balance, ml	Median (Q_1_–Q_3_)	3310 (2635–4860)
Serum creatinine at admission to ICU, mg/dl	Median (Q_1_–Q_3_)	0.85 (0.77–1.01)
Serum creatinine after 24 h, mg/dl	Median (Q_1_–Q_3_)	1 (0.85–1.12)
Duration of mechanical ventilation, h	Median (Q_1_–Q_3_)	17.75 (14.25–19.8)
Length of stay on ICU, h	Median (Q_1_–Q_3_)	23.9 (21.3–69.6)
Length of stay in hospital, d	Median (Q_1_–Q_3_)	14 (9–17)
CPR	Number (percentage)	0 (0)
Revision surgery	Number (percentage)	0 (0)

*NYHA* New York Heart Association classification, *PAOD* peripheral arterial occlusive disease, *CPB* cardiopulmonary bypass, *RBC* red blood cell, *PaO*_*2*_ arterial oxygen partial pressure, *FiO*_*2*_ inspiratory oxygen fraction, *ICU* intensive care unit, *Hgb* hemoglobin, *CPR* cardiopulmonary resuscitation, *Q*_*1*_ *–Q*_*3*_ percentile 25–75

### CABG impairs angiopoietin balance

**Fig. 2:**
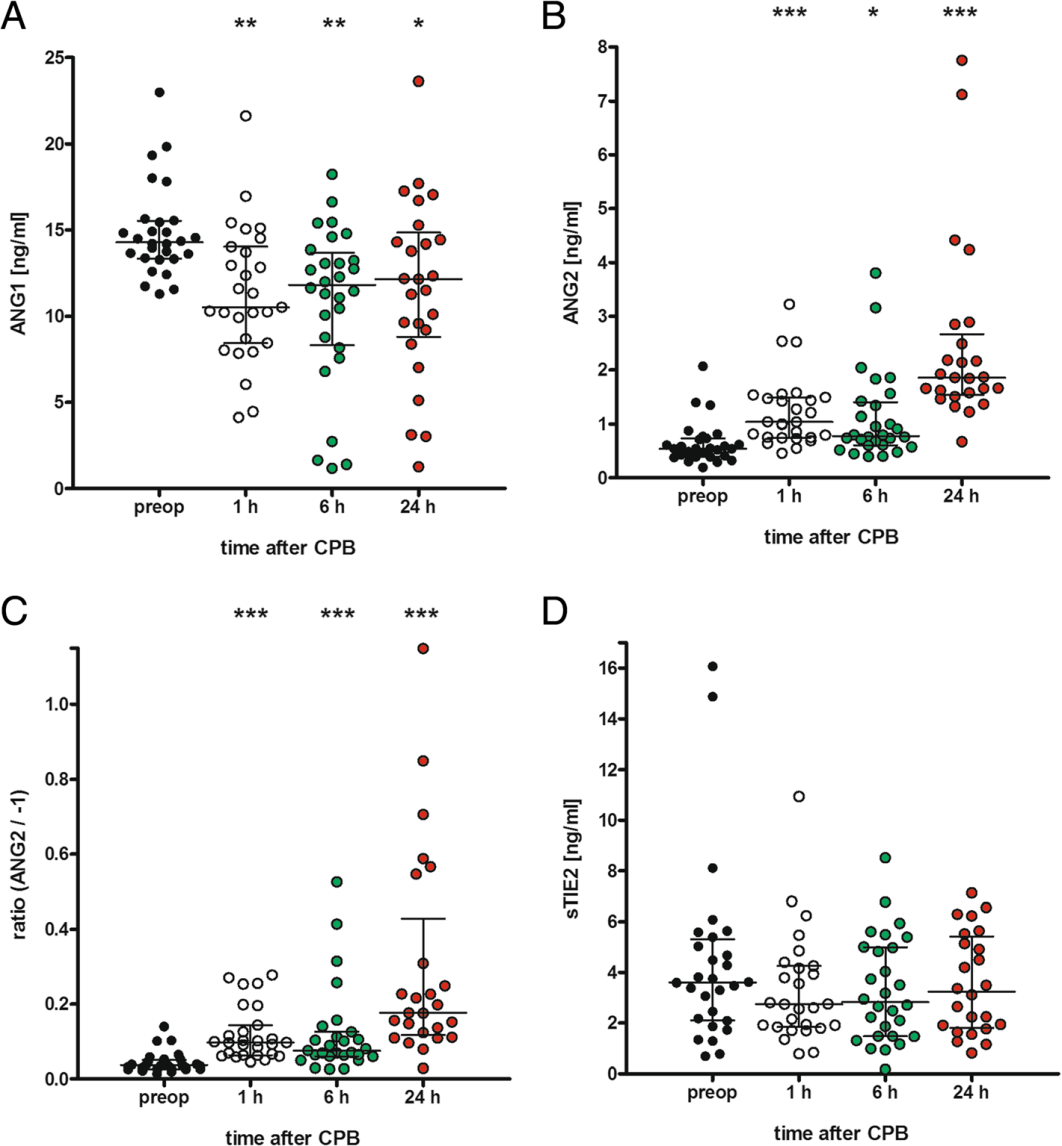
Changes in angiopoietin and TIE2 serum levels upon coronary artery bypass graft (CABG). Serum was sampled from patients being scheduled for elective on-pump CABG before surgery (*preop*) and 1, 6 and 24 h after closure of the aortic clamp. Serum was analyzed for angiopoietin 1 (*ANG1*) (**a**), ANG2 (**b**) and soluble TIE2 receptor (*sTIE2*) (**d**) by enzyme-linked immunosorbent assay. Furthermore, the ANG2/ANG1 ratio was calculated (**c**). N = 28 (24-h time point, n = 26), median with percentile 25–75, *p <0.05, **p <0.01, ***p <0.001 (vs. preop). *CPB* cardiopulmonary bypass

CABG induced an immediate and persistent decrease in ANG1 and increase in ANG2 serum levels. Median ANG1 levels significantly decreased 1 h after aortic de-clamping compared to pre-surgical concentrations (10.5 vs. 14.3 ng/ml, *p* <0.01), and the decrease was sustained for 24 h (12.2 ng/ml; Fig. 2a). In contrast, circulating ANG2 levels increased significantly 1 h after aortic de-clamping compared to pre-surgical concentrations (1.04 vs. 0.54 ng/ml ng/ml, *p* <0.001), and the increase was sustained during the 24-h observational period (1.86 ng/ml; Fig. 2b). The ratio between ANG2 and ANG1 at baseline was 0.04. This ratio significantly increased to 0.1 after 1 h and continued up to 0.18 at 24 h after CABG (*p* <0.001), indicating a robust five-fold increase 24 h after aortic de-clamping (Fig. 2c).

The soluble form of the TIE2 receptor (sTIE2) was detectable in the serum (baseline level 3.61 ng/ml); however, CABG had no effect on sTIE2 serum concentrations (Fig. 2d).

### Clinical parameters correlate with changes in ANG2/ANG1 ratio

**Fig. 3:**
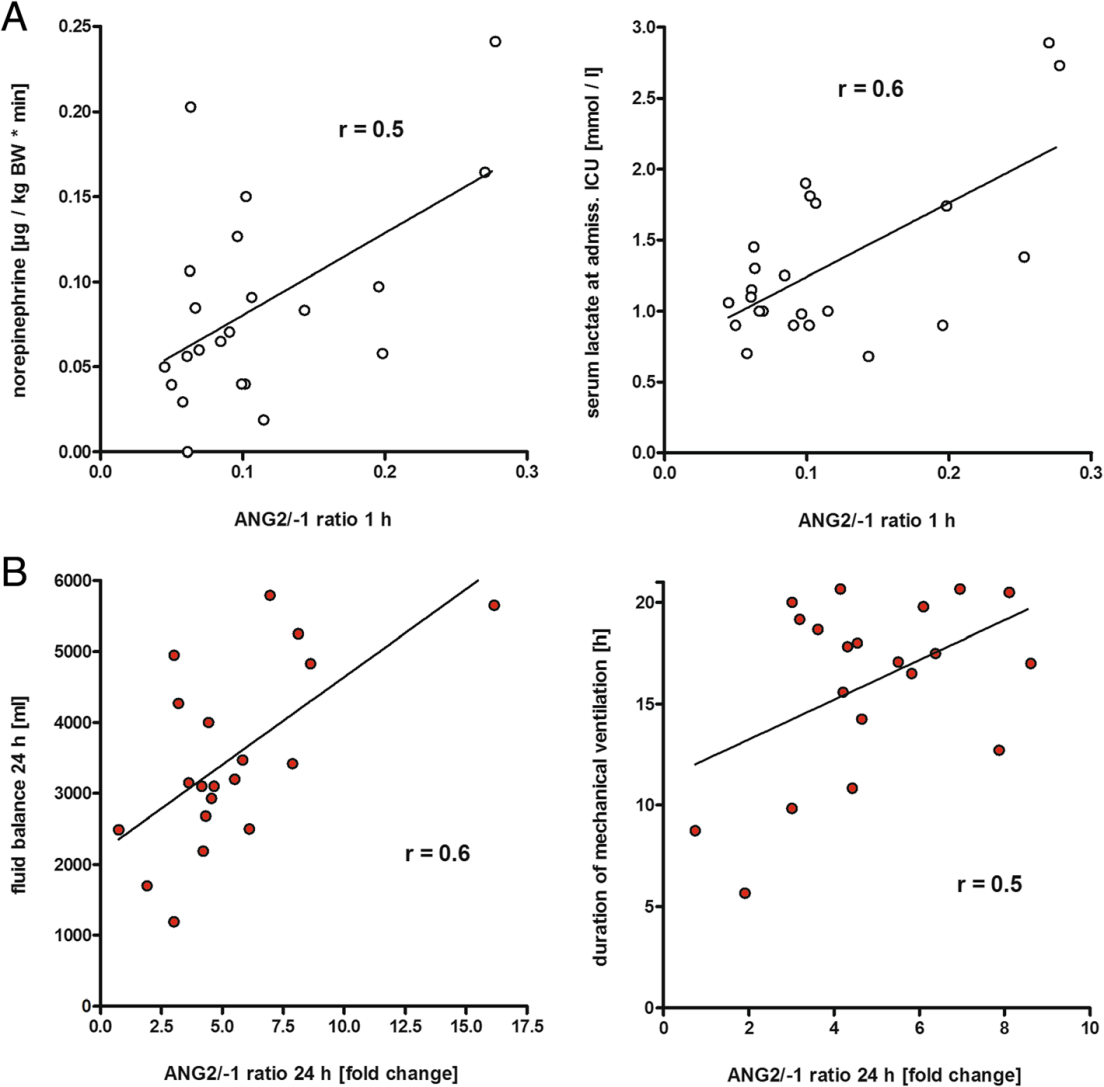
Correlation between the angiopoietin (*ANG*)2/ANG1 ratio and clinical parameters. Pearson’s correlation was calculated to test the association between altered ANG2/ANG1 balance and clinical parameters. **a** Correlation between early ANG imbalances (1 h after coronary artery bypass graft (CABG)) and dosage of norepinephrine administered after CABG (*left*), and serum lactate levels measured at admission to the ICU (*right*). **b** Correlation between late increase of ANG balance (24 h after CABG) and crystalloid fluid balance 24 h after admission to ICU (*left*), and duration of mechanical ventilation (*right*). *BW* body weight

Correlation analysis revealed an association between an increased ANG2/ANG1 ratio and several clinical parameters. The ANG2/ANG1 ratio at 1 h after aortic de-clamping was positively correlated with the duration of ischemia and reperfusion (*r* = 0.5, *p* <0.05) and the dosage of administered vasopressive agents (*r* = 0.5, *p* <0.01; Fig. 3a). Furthermore, serum lactate as a surrogate marker for impaired tissue oxygenation was likewise positively associated with early angiopoietin imbalance (*r* = 0.6, *p* <0.005). Of note, positive correlations with increased serum lactate levels remained detectable up to 24 h after CABG (*r* = 0.7, *p* <0.005).

An increased ANG2/ANG1 ratio 24 h after CABG correlated with a positive balance of crystalloid infusion fluids (*r* = 0.6, *p* <0.005; Fig. 3b). Patients with ANG2/ANG1 values above the median had significantly greater fluid intake than those with ratios below the median and a trend towards longer ICU LOS (Table 2). Moreover, an increase in the angiopoietin ratio after 6 h and after 24 h, was associated with the duration of mechanical ventilation, possibly reflecting vascular leakage-related impaired pulmonary performance (*r* = 0.5, *p* <0.05; Fig. 3b).

**Table 2:** Influence of the 24-h ANG2/ANG1 ratio

Variable	ANG2/ANG1	ANG2/ANG1	*p* value
	<0.18	>0.18	
Details of patients and procedure			
Preoperative ejection fraction, %	60 (53–67)	50 (44–55)	0.01
CPB time, minutes	113 (90–125)	110 (103–125)	0.49
Aortic cross-clamp/reperfusion time, minutes	105 (85–120)	105 (93–115)	0.9
Postoperative details (T_24_)			
PaO_2_/FiO_2_ ratio	407 (360–502)	429 (182–481)	0.79
Lactate, mmol/l	1.27 (1–1.4)	1.32 (1.1–1.2)	0.68
Hgb, mmol/l	9.7 (9.3–10.4)	10.2 (9.1–11.6)	0.52
Crystalloid fluid balance, ml	3100 (2340–3735)	4830 (3310–5720)	0.01
Serum creatinine, mg/dl	1.03 (0.9–1.12)	0.98 (0.74–1.19)	0.58
Duration of mechanical ventilation, h	16.5 (10.3–18.9)	18.8 (17.1–20.2)	0.12
ICU LOS, h	22.2 (21–35.4)	44.4 (21.3–74.6)	0.2
LOS hospital, days	14 (9–15)	14 (11–21)	0.12

The study population was divided into two groups according to the angiopoietin (*ANG*)2/ANG1 ratio measured 24 h after aortic de-clamping using the overall median ratio (0.18) as threshold value. Details are presented for patients, procedures, and postoperative variables recorded 24 h after admission to ICU (*T*_*24*_). Median with percentile 25–75, two-tailed Mann–Whitney test. *CPB* cardiopulmonary bypass, *PaO*_*2*_ arterial oxygen partial pressure, *FiO*_*2*_ inspiratory oxygen fraction, *Hgb* hemoglobin, *ICU LOS* length of stay on intensive care unit, *LOS hospital* length of stay in hospital

The associations were more modest or even unverifiable when testing correlation independently with ANG1 or ANG2.

### An increased ANG2/ANG1 ratio facilitates endothelial hyper-permeability ex vivo

**Fig. 4:**
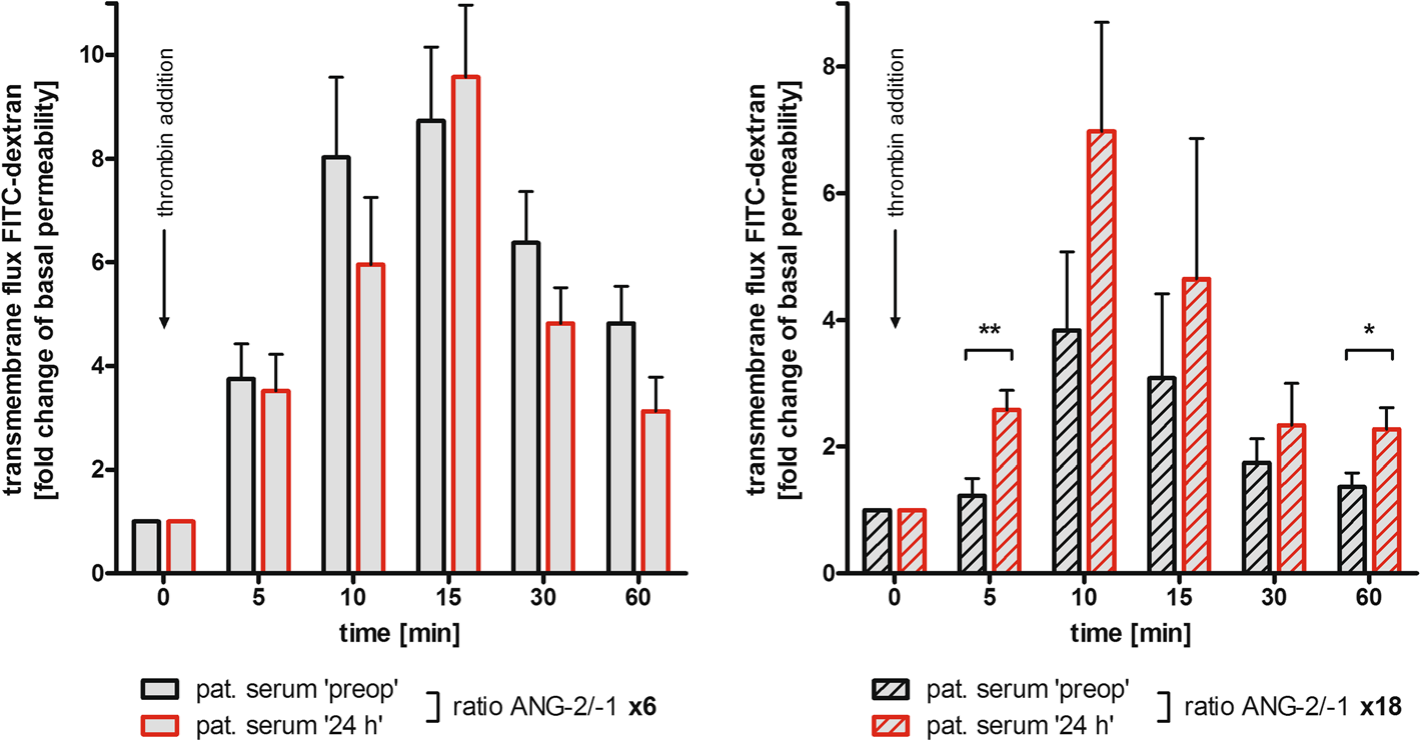
Influence of angiopoietin (*ANG*)2/ANG1 imbalance on endothelial hyper-permeability ex vivo*.* Human pulmonary microvascular endothelial cells were seeded at high density on gelatin-coated permeable supports and cultured to form a confluent monolayer. After pre-incubation with filtered and diluted (7.5 %) patient serum (preoperative (*preop*) or 24 h, respectively) for 30 minutes, fluorescein isothiocyanate (*FITC*)-conjugated dextran (70 kDa, final concentration 1 mg/ml) was added to the upper compartment and the inserts were set on a magnetic stirrer. Thrombin (final concentration 1 IU/ml) was added to induce endothelial permeability. Samples (of 50 μl) were taken from the lower compartment at the indicated time points. Dextran concentration was determined using a fluorescent plate reader. Thrombin-induced hyper-permeability is expressed as fold of the average basal flux during the initial 15-minute equilibration period before thrombin addition. *Left*, change in endothelial cell (EC) permeability after pre-incubation with serum samples with a mild shift in the ANG2/ANG1 ratio upon coronary artery bypass graft (CABG) (6-fold from baseline to 24 h). *Right*, change of EC permeability after pre-incubation with serum samples with pronounced shift in the ANG2/ANG1 ratio upon CABG (18-fold from baseline to 24 h). N = 8–10, mean ± standard error of the mean, *p <0.05, **p <0.01. *Pat* patient

Thrombin induced rapid transendothelial dextran flux within 10 to 15 minutes (Fig. 4). Thereafter, with resealing of ECs, the transmembrane flux decreased, reaching baseline levels after approximately 60 minutes. Pre-incubation of ECs with serum samples containing a mild shift in angiopoietins upon CABG (increase of ANG2/ANG1 ratio 6-fold from baseline to 24 h) had no significant effect on thrombin-induced permeability compared to the use of preop serum. However, serum samples with a pronounced ANG2/ANG1 imbalance upon CABG (increase of ANG2/ANG1 ratio 18-fold from baseline to 24 h) provoked a significant hyper-permeability of the endothelial monolayer upon thrombin addition in comparison to preop samples. This effect was maintained over at least 60 minutes.

### CABG increases IL-6 levels

**Fig. 5:**
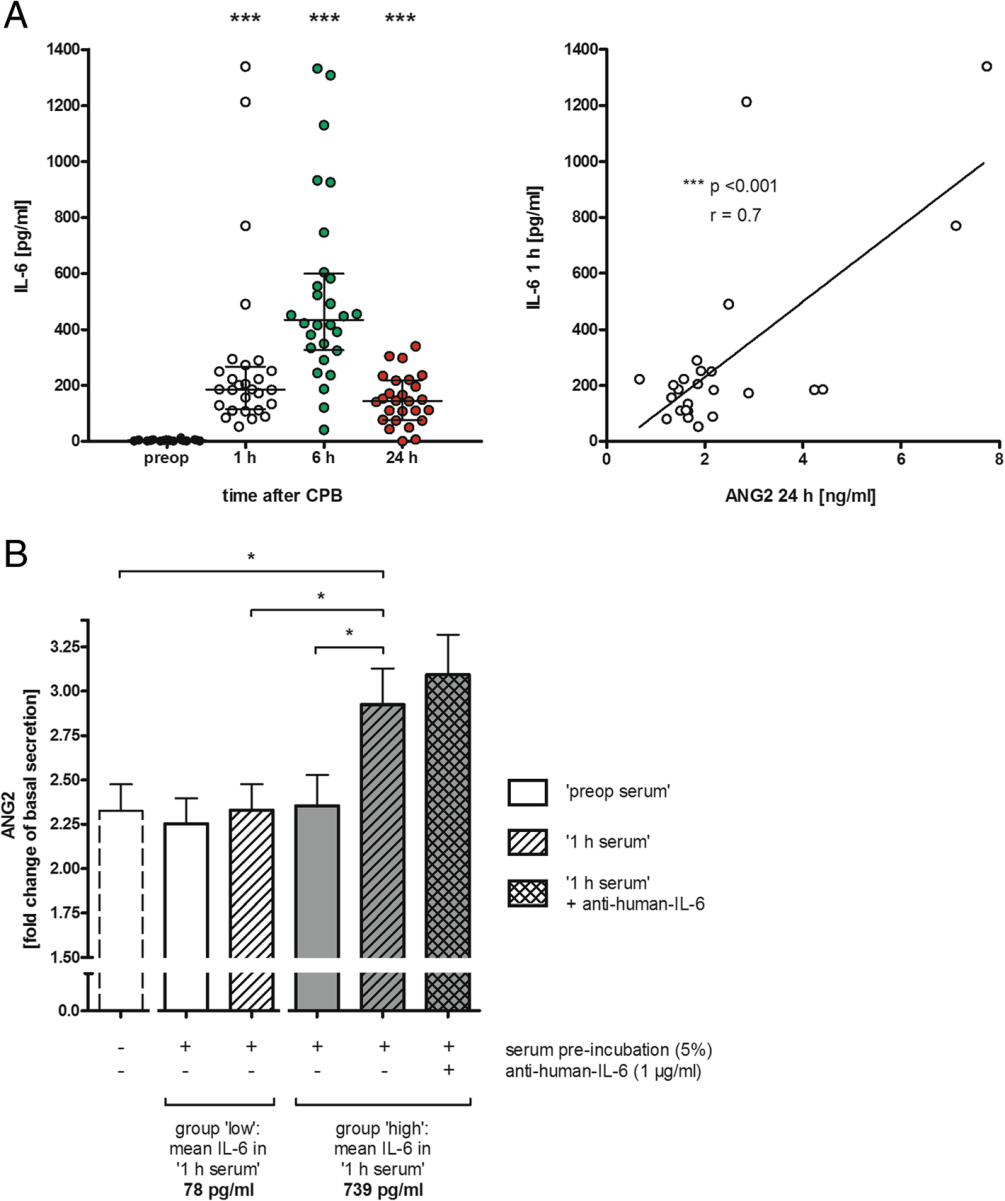
Association between interleukin (*IL*)-6 serum activity and angiopoietin (*ANG*)2 levels. **a** Serum was sampled from patients being scheduled for elective on-pump coronary artery bypass graft (CABG) before surgery (*preop*) and 1, 6 and 24 h after closure of the aortic clamp. Serum was analyzed for IL-6 (enzyme-linked immunosorbent assay (ELISA)) (*left*). The early increase in IL-6 1 h after CABG correlates with further increasing ANG2 levels measured 24 h after de-clamping (*right*): n = 28 (24 h time point, n = 26), median with percentile 25–75, ***p <0.001 (vs. preop). **b** Human pulmonary microvascular endothelial cells were cultured in 96-well plates and pre-incubated with filtered and diluted (5 %) patient serum (preop (*clear bars*) or 1 h (*hatched bars*), respectively) and anti-human-IL-6 antibody (1 μg/ml) for 24 h. Supernatant was discarded, and triggered ANG2 secretion was induced by adding thrombin (1 IU/ml) in fresh culture medium. After 1 h, the ANG2 concentration was determined by ELISA. Fold change of triggered ANG2 secretion (compared to basal secretion without thrombin addition) is shown after pre-incubation with serum with low IL-6 activity (*white bars*) vs. serum with high IL-6 concentration (*gray bars*). The bar on the *right* shows ANG2 secretion when ‘high IL-6 1 h’ serum pre-incubation was combined with anti-human-IL-6 antibody: n = 6–9, mean ± standard error of the mean, *p <0.05. *CPB* cardiopulmonary bypass

SIRS after CABG is characterized as a systemic pro-inflammatory reaction accompanied by endothelial barrier dysfunction and vasoplegia. Pre-surgical IL-6 levels were low (3.0 pg/ml, Fig. 5a); however, upon CABG, IL-6 concentrations significantly increased 1 h after aortic de-clamping and were even further increased after 6 h (435 pg/ml, *p* <0.001). At 24 h after aortic de-clamping, IL-6 levels remained significantly elevated compared to pre-surgical levels (144 pg/ml, *p* <0.001). Neither TNF-α nor IL-1β was detectable in the serum before or after CABG (data not shown).

### Pro-inflammatory conditioned serum, but not IL-6, increases ANG2 release ex vivo

The early increase in IL-6 serum concentrations 1 h after de-clamping was associated with increasing ANG2 levels measured 24 h after de-clamping (*r* = 0.7, *p* <0.001; Fig. 5a). The ANG2/ANG1 imbalance upon CABG is mainly driven by increasing serum ANG2 levels (Fig. 2a). To test the hypothesis that IL-6 is a humoral factor enhancing the release of ANG2 from WPBs, HPMECs were incubated with patient serum (preop or 1 h, respectively) with and without addition of an inhibiting anti-human-IL-6 antibody, followed by the detection of released ANG2 in the supernatant. Pre-incubation with either preop or with 1-h serum had no significant effect on ANG2 secretion when the IL-6 content of the sample was low (on average 78 pg/ml in the 1-h serum; Fig. 5b). In contrast, pre-incubation with 1-h serum samples with nearly 10 times higher IL-6 contents (739 pg/ml) induced a significant amplification of ANG2 activity in the cell supernatant compared to incubation with preop serum from the same patients. Addition of an inhibiting anti-IL-6 antibody did not abolish this effect.

## Discussion

The objective of this study was to assess the impact of CAGB surgery-induced alterations in ANG1, ANG2 and TIE2 levels on post-surgical SIRS and to evaluate potential mechanisms inducing capillary leakage. Our results indicate that CABG induced profound changes in the ANG2/ANG1 balance with loss of the barrier-protective ANG1 and an increase in the barrier-disruptive ANG2 contents in the patients’ serum. This imbalance was observed in adult patients with systemic pro-inflammatory response characterized by elevated IL-6 levels. The molecular changes correlated with clinical surrogate parameters of an impaired endothelial barrier function, such as fluid balance, application of vasoconstrictors, and prolonged ventilation.

In healthy individuals, constitutive expression of ANG1 results in much higher serum concentrations than ANG2, which is mainly secreted upon endothelial stimulation; therefore, an increased ratio between ANG2 and ANG1 (in healthy adults a numerical value below 1) serves as marker of endothelial activation [23]. Various cardiovascular diseases including atherosclerosis, CAD, and acute myocardial infarction (MI) affect the expression of angiopoietins [27, 28]. Upon clinical manifestation of these conditions, serum levels of ANG2 increase significantly and its biological effect is to overcome the basal vascular-stabilizing function of ANG1 [19, 23]. The population in the current study comprised clinically stable patients being scheduled for elective CABG, thus basal ANG2/ANG1 ratios were marginally elevated.

ANG2/ANG1 ratios increased early after CABG in our patients and remained elevated for 24 h. Giuliano et al. described similar alterations in angiopoietin serum levels in children undergoing surgery for congenital heart disease [29]. However, in infants with congenital heart disease, systemic hypoxia due to veno-arterial shunts induces significant changes in angiopoietin levels, which persist into later life, limiting the relevance of these results to our adult patients [30]. Furthermore, ANG2 response to cardiac surgery may be age-dependent in general [29].

The observed alterations in the ANG2/ANG1 balance correlated with several clinical variables. Besides the surgical trauma, ischemia and reperfusion (I/R) are among the most harmful events during cardiac surgery, occurring locally in the heart during cross-clamping and systemically following the reperfusion period. The myocardium itself is a major source of CPB-related inflammation, and extended cross-clamp times are associated with increased cytokine expression [31, 32]. Beside I/R-mediated pro-inflammatory responses, I/R also contributes to alterations in angiopoietin balance, favoring ANG1 downregulation, and increasing ANG2 activity [33]. Accordingly, a shift in the ANG2/ANG1 ratio after CABG was associated with I/R time in our patients.

The observed correlation beteen an altered ANG2/ANG1 ratio and the dosage of administered vasopressive agents offers scope for speculation. On one hand, it has been shown that loss of the balance between vaso-protective ANG1 and vaso-destructive ANG2 (e.g., during sepsis) results in the emergence of endothelial dysfunction [16]. The latter is described as the lack of constitutive endothelial NO synthase (eNOS), being outnumbered by inducible vaso-relaxing factors (such as reactive oxygen species or inducible NOS (iNOS)) [34]. This may lead to profound vasoplegia with an increased need for vasopressive agents [35]. On the other hand, we noted that disturbances in the angiopoietin balance were furthermore associated with elevated serum lactate levels throughout the entire observation period. Lactate is increasingly produced when perfusion is insufficient to meet the oxygen demand of the cell, and tissue hypo-perfusion, and thus, ischemia contribute to both lactic acidosis and shifts in the ANG2/ANG1 ratio [33]. Vasopressive agents themselves, such as norepinephrine, provoke tissue hypo-perfusion and thus, increased lactate production and ANG2 release [36]. Interestingly, lactate itself stimulates the upregulation of ANG1 and subsequently activates TIE2 signaling, and may therefore be a compensatory response to counteract impaired microcirculation [37].

Contact between blood and artificial surfaces may alter the expression of ANG2 [38]. However, ECs release the previously synthesized protein from cytoplasmatic vesicles upon a distinct trigger, and it remains unclear what finally mediates ANG2 release during and after CABG. In our study, elevated serum ANG2, mainly driving the shift in the ANG2/ANG1 balance upon CABG, was correlated with an early increase in IL-6 serum levels. Results from clinical studies suggest that pro-inflammatory mediators may be associated with the regulation of ANG2 [39, 40]. IL-6 is not known to trigger WPB exocytosis. However, signal transducer and activator of transcription 3 (STAT3) as a key regulator of the IL-6 receptor pathway has been shown to upregulate ANG2 synthesis upon phosphorylation [41]. Thus, our hypothesis was that IL-6 itself upregulates ANG2 synthesis and induces increased filling of the vesicles, subsequently leading to enhanced exocytosis. Sera with high IL-6 levels 24 h after CABG significantly increased the amount of ANG2 in the supernatant of ECs compared to low-level IL-6 sera. However, blocking experiments revealed that IL-6 is merely the reflection of a strong pro-inflammatory response to CABG and does not significantly influence the EC independently. The mechanisms leading to increased ANG2 serum activity upon CABG remain to be further investigated.

Elevated ANG2/ANG1 ratios are related to vascular leakage [24]. When we treated confluent layers of ECs with patient sera, we observed a pronounced disruption of the endothelial barrier for high molecular weight dextran upon thrombin addition, and this effect was dependent upon the extent of the ANG2/ANG1 imbalance. Compared to preop sera, 24-h sera with a distinct shift in the ANG2/ANG1 ratio after CABG provoked significant hyper-permeability of endothelial monolayers. Unfortunately, an inhibiting ANG2 antibody was not available for us, thus, our study lacks the final causal proof. However, despite this limitation, there is strong evidence for the contribution of altered ANG2/ANG1 balance to endothelial barrier dysfunction after CABG, likewise supported by other authors. Clajus et al. demonstrated increasing ANG2 serum levels during and after cardiac surgery [42]. Incubating ECs with these sera induced alterations of the endothelial architecture. As this effect was abolished by the addition of recombinant ANG1, the authors concluded that the observed EC permeability was ANG2-mediated. Clinically, in our study, angiopoietin ratios measured later during the clinical course were correlated with positive fluid balance as a surrogate marker for endothelial barrier disruption. Moreover, high ANG2/ANG1 levels were associated with prolonged duration of mechanical ventilation. This is likewise in line with reports from other authors describing a key role of angiopoietins for the development of sepsis-induced vascular leakage and pulmonary dysfunction [14–16, 25]. Recently, Uchida et al. demonstrated an association between elevated postoperative ANG2 levels and the development of respiratory failure after cardiac surgery [43]. However, we can only partially confirm this, because the ANG2 levels that we measured after CABG were not correlated with a prolonged need for mechanical ventilation (in contrast to the ANG2/ANG1 ratio).

Our study generates hypotheses, but it has limitations. First, due to a small sample size and strict inclusion and exclusion criteria, it lacks external validity. Second, we included neither a healthy control group (to correctly classify baseline angiopoietin levels) nor patients undergoing surgery using modified CPB or even off-pump CABG surgery. Thus, the observed effects cannot be exclusively ascribed to CPB. Recently, Uchida et al. reported significantly altered angiopoietin serum levels in patients undergoing on-pump vs. off-pump cardiac surgery [43]. Elevated ANG2 and decreased ANG1 levels after percutaneous coronary revascularization suggest that the heart is likewise a major source of the altered ANG2/ANG1 ratio even after CABG [44]. However, surgical trauma may be another cause of elevated ANG2 levels, as evidenced by studies on patients after traumatic lung injury or non-cardiac thoracic surgery [45, 46]. Reducing invasiveness by the use of endoscopic techniques likewise reduces post-procedural ANG2 serum levels in those patients, which furthermore supports the hypothesis that CPB during cardiac surgery may play a subordinate role in influencing the ANG2/ANG1 balance compared to the surgical trauma [47]. Our data support this conclusion as well, because total CPB time did not correlate with altered ANG2/ANG1 ratio. Third, the cohort we studied consisted of elective low-risk patients who could be rapidly discharged from the ICU. In high-risk patients, the postoperative levels of circulating angiopoietins may be different. Fourth, endothelial permeability was assessed in vitro using a single cell type assay, which is rather artificial. Fluid balance and duration of mechanical ventilation are only clinical surrogates for a disturbed endothelial barrier. Methods to evaluate vascular leakage in vivo, such as measuring extravascular lung water, bioelectrical impedance analysis to determine the extracellular and total body water, or imaging of extravasated radiolabeled macromolecules, would have been advantageous [48, 49]. Finally, there was a lack of an inhibiting ANG2 antibody to prove a causal relationship between increased ANG2/ANG1 balance and endothelial hyperpermeability.

## Conclusions

The results of our study provide evidence that CABG significantly affects the balance between ANG1 and ANG2 towards a dominance of the barrier-disruptive ANG2. Our data suggest that this ANG2/ANG1 imbalance contributes to an increased postoperative endothelial permeability, which is likewise reflected by the further clinical course. This study is in line with others describing dynamic changes in angiopoietins during cardiac surgery, however, it is the first to strongly suggest a biological effect on endothelial permeability. The mechanisms leading to altered angiopoietin activity remain to be investigated in future studies.

## Key messages

- CABG surgery shifts the balance between ANG1 and ANG2 towards a dominance of the barrier-disruptive ANG2.
- This contributes to surrogate markers of increased postoperative endothelial permeability
- The increased serum ANG2/ANG1 ratio facilitates endothelial hyper-permeability ex vivo
- Systemic inflammation after CABG correlates with increasing serum ANG2 levels
- Pro-inflammatory conditioned patient serum contributes to enhanced endothelial ANG2 release ex vivo
